# Clinical Impact of FDG PET/CT in Pulmonary Nodule Characterization: Current Perspectives on Dual-Time-Point Imaging and Semi-Quantitative Imaging Metrics

**DOI:** 10.3390/cancers17203353

**Published:** 2025-10-17

**Authors:** Nikolaos Kapsoritakis, Foteini Tsitoura, Maria Stathaki, Olga Bourogianni, Panagiotis Georgoulias, Georgios D. Barmparis, Antonios Bertsias, Giorgos P. Tsironis, Sophia Koukouraki

**Affiliations:** 1 Department of Nuclear Medicine, Medical School, University of Crete, 715 00 Heraklion, Greece; mariast_cha@yahoo.gr (M.S.); ompourogianni@yahoo.gr (O.B.); koukour@uoc.gr (S.K.); 2 Department of Physics, University of Crete, 700 13 Heraklion, Greece; f.tsitoura@gmail.com (F.T.); barmparis@physics.uoc.gr (G.D.B.); gts@physics.uoc.gr (G.P.T.); 3 Department of Nuclear Medicine, University General Hospital of Larissa, Faculty of Medicine, University of Thessaly, 413 34 Larisa, Greece; pgeorgoul@uth.gr; 4 Clinic of Rheumatology and Clinical Immunology, Medical School, University of Crete, 715 00 Heraklion, Greece; antonisbertsias@yahoo.gr

**Keywords:** 18F FDG, PET/CT, pulmonary nodules, SUV, MTV, TLG, DTPI, AI, PET radiomics

## Abstract

**Background/Objectives:** Pulmonary nodules (PNs) are a common incidental finding on conventional imaging. Differentiating benign from malignant lesions remains a diagnostic challenge. 18F-fluorodeoxyglucose positron emission tomography/computed tomography (18F-FDG PET/CT) has become an essential imaging modality in this setting. This review aims to evaluate the clinical impact of PET/CT parameters and techniques, focusing on semi-quantitative imaging biomarkers and dual-time-point imaging. **Methods:** This review is organized into three main sections. First, qualitative analysis and PET key metrics are analyzed, including standardized uptake value (SUVmax), metabolic tumor volume (MTV), and total lesion glycolysis (TLG) indices, highlighting their diagnostic and prognostic significance. The second section focuses on the clinical utility of dual-time-point imaging (DTPI), evaluating its ability to differentiate between benign and malignant PNs through changes in SUV over time (ΔSUVmax). We compare these advanced imaging approaches with histopathological diagnosis, the current gold-standard method, highlighting the potential of advanced PET/CT techniques in clinical decision-making. The last section focuses on future applications of PET/MR, artificial intelligence, and PET radiomics. **Results:** Evidence indicates that high SUV, MTV, and TLG values are significantly associated with malignant PNs and aggressiveness. Moreover, DTPI with ΔSUV, ΔMTV, and ΔTLG further enhances specificity and accuracy in characterizing PNs. Despite a lack of standardization, studies have shown better accuracy when advanced PET/CT parameters are used. **Conclusions:** While DTPI and semi-quantitative PET parameters are not yet universally adopted in daily clinical practice, evidence supports their role in enhancing the characterization of indeterminate PNs. More prospective studies are needed.

## Simple Summary

Pulmonary nodules are frequently detected via conventional imaging, and distinguishing benign from malignant lesions remains a diagnostic challenge. 18F-FDG PET/CT has become a key imaging modality for evaluating these nodules based on their metabolic activity. This review explores the clinical impact of FDG PET/CT in characterizing pulmonary nodules, focusing on dual-time-point imaging and semi-quantitative metrics such as standardized uptake values (SUVs), evolving metrics including metabolic tumor volume (MTV) and total lesion glycolysis (TLG), and future perspectives including artificial intelligence (AI) and PET radiomics. Dual-time-point imaging improves diagnostic accuracy by assessing changes in FDG uptake over time, while semi-quantitative analysis provides measurements to support clinical decision-making. We highlight the current limitations, recent advances, and potential future applications of these techniques in improving diagnostic accuracy.

## 1. Introduction

Lung cancer is a major public health issue and the leading cause of cancer-related life-threatening incidents and mortality worldwide, according to the World Health Organization (WHO). Pulmonary nodules (PNs) are defined as intrapulmonary focal lesions measuring 0.5–3 cm in diameter [1,2]. The ability to characterize PNs as benign or malignant remains a critical challenge in clinical oncology due to the variety of potential causes, such as benign inflammatory lesions and metastatic and primary lung malignancies [1].

PNs are assessed on CT scans according to morphological characteristics such as size, shape, and density, among which. size is the primary indicator of malignancy risk. The larger a nodule is (typically, >8 mm is the cut-off point), the higher the probability of malignancy.

PNs measuring 5–10 mm have a malignant potential of approximately 6%, while those of 20–30 mm can be of malignant origin in up to 64% of patients. The malignancy rates of nodules detected incidentally during screening procedures ranges from 3% to 60% [2].

Shape also plays an important role, as irregular or spiculated nodules with finger-like projections are suspicious than those with smooth and round borders. Additionally, PNs can be classified into three subgroups based on density, i.e., solid, sub-solid, and ground glass, with each exhibiting different malignancy risk. Solid PNs generally have a higher malignant potential than sub-solid and ground-glass nodules. Furthermore, the growth rate of a lesion is another parameter in the characterization of PNs, where a faster growth rate is associated with higher malignant potential [3].

PET/CT is a hybrid imaging technique that combines positron emission tomography (PET) and computed tomography (CT), providing both functional and anatomical information. FDG is a radiolabeled glucose analogue, transported into cells via glucose transporters (primarily GLUT1 and GLUT3) and subsequently phosphorylated by hexokinase to FDG-6-phosphate. Unlike glucose, FDG-6-phosphate is not further metabolized through glycolysis and becomes metabolically trapped within the cell. Both malignant and inflammatory cells show increased glucose metabolism [4].

18F FDG PET/CT is widely used to assess solitary or multiple PNs using both qualitative and semi-quantitative analysis, enhancing the accuracy, diagnostic evaluation, staging, and therapeutic management of patients with PN. European guidelines from the Fleischner Society and the European Respiratory Society (ERS) recommend 18F FDG PET/CT for PNs larger than 8 mm [5].

## 2. Materials and Methods

**Figure 1:**
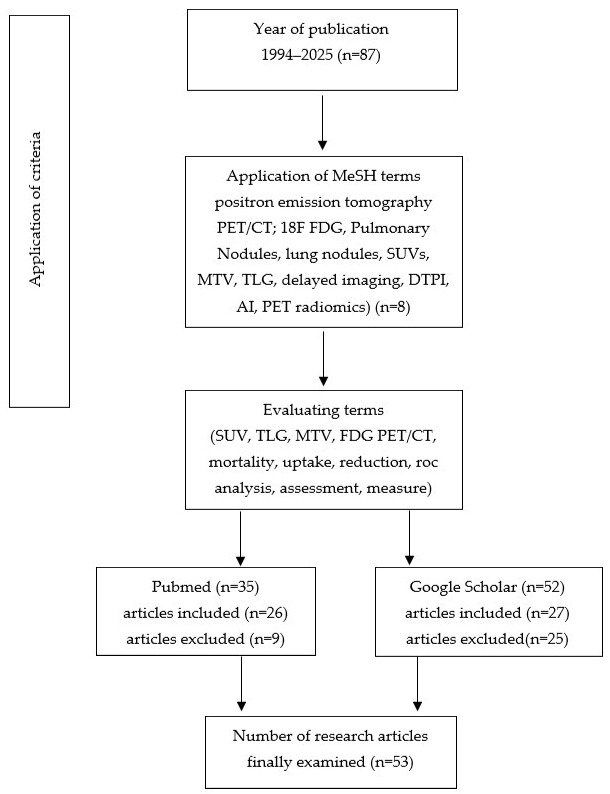
Study selection flow diagram.

A search of the literature published from 1994 to 2025 was performed to identify relevant studies, investigating the role of 18F FDG PET/CT in the characterization of PNs. Keywords and MeSH terms included imaging modality positron emission tomography or PET/CT; radiotracer 18F Fluorodeoxuglucose or 18F FDG; dual-time-point imaging (DTPI) or delayed imaging as an imaging technique; quantitative parameters including SUVs, MTV, and TLG metrics; and the clinical question of determining pulmonary nodules or lung nodules (Figure 1). The search was based on databases including PubMed, with Google Scholar as a supplementary source.

Regarding inclusion criteria, we considered original research studies (prospective and retrospective) evaluating the quantitative metrics of SUVs, MTV, TLG, and DTPI FDG PET/CT in PNs; human studies reporting the evaluation of PNs in early- and delayed-phase FDG PET/CT published in English; and studies reporting diagnostic accuracy, changes in SUV, or clinical outcomes related to dual-time-point imaging, and pulmonary nodules of less than 3 cm.

Case reports and letters, studies unrelated to pulmonary nodules or lung cancer, articles lacking sufficient methodological or outcome details, and studies related to pulmonary masses of size larger than 3 cm were excluded (Table 1).

## 3. Results

### 3.1. Qualitative Analysis

Qualitative assessment of 18F FDG PET/CT imaging is based on visual evaluation of radiotracer uptake in PNs, which is graded as no uptake, low, intermediate, or high compared to the mediastinal blood pool (MBP). Assessment integrates radiotracer uptake patterns from PET (focal, diffuse, homogeneous, heterogeneous, peripheral, or eccentric uptake) and morphological patterns from CT (size, shape, margins, and density). Different types of radiotracer uptake have different clinical meanings. Higher uptake is correlated with higher probability of malignancy. A low or negative uptake does not exclude malignancy. According to Corica F et al., only 20% of PNs with low or absent 18F FDG uptake were malignant, while malignancy rates increased to 45% and 90% in moderate- and intense-uptake PNs, respectively [28]. Although this method remains subjective, it continues to play a crucial role in staging and treatment planning.

### 3.2. Semi-Quantitative Assessment

Semi-quantitative 18F FDG PET/CT analysis quantifies the metabolic activity of lesions compared to background or a reference organ. This method provides additional data to visual assessment [39]. The most widely used semi-quantitative parameters are the standardized uptake values (SUVs), particularly the maximum standardized uptake value (SUVmax) and mean standardized uptake value (SUVmean).

However, these parameters can be unreliable for small nodules (<0.5 cm) due to the partial volume effect, in addition to in malignancies with a low glycolytic rate. Furthermore, 18F FDG PET/CT has limited sensitivity in the assessment of ground-glass opacity and semi-solid PNs as they show low glycolytic activity resulting in reduced FDG uptake and a higher rate of false-negative results. To overcome these limitations, strategies such as dual-time-point imaging (DTPI); emerging quantitative PET biomarkers including metabolic tumor volume (MTV) and total lesion glycolysis (TLG); and artificial intelligence (AI), radiomics, and hybrid PET/MRI models are currently under investigation [29,30,40].

### 3.3. Semi-Quantitative Metrics—SUVs

There are several types of SUVs based on the method of normalization and measurement. For normalization, body weight SUV (SUVbw) is mostly used, normalized by a patient’s body weight, but it can overestimate obese patients. Lean body mass SUV (SUVlbm) is more accurate and recommended for oncological patients according to PERCIST criteria. Body surface area SUV (SUVbsa) can be normalized by body surface area but is rarely used in clinical practice. For the measurement approach, SUVmax is the most frequently used parameter reflecting the maximum pixels within a region of interest (ROI). It reflects the radiotracer uptake normalized to the injected dose and the patient’s body parameters. It is calculated by creating a region of interest (ROI) around the lesion [7]. Traditionally, a threshold of SUVmax > 2.5 is considered indicative of malignancy [8]. However, SUVmax is affected by several factors including the shape of the ROI, nodule sizes of <1 cm (partial volume effect), scanner parameters, acquisition protocols, and patient body mass index (BMI) [41].

A tumor’s characteristics and metabolic profile affect the evaluation of SUVmax and influence 18F FDG uptake. Well-differentiated and slow-growing PNs tend to have lower 18F FDG uptake and as a result reduced SUVmax values, whereas aggressive lesions exhibit higher 18F FDG uptake and increased SUVmax values. Infectious or inflammatory PNs often have elevated SUVmax values, comparable to malignant lesions, making interpretation challenging. According to Shin et al., qualitative assessment of PNs provides higher sensitivity and specificity than using only SUVmax, especially in small PNs (<0.5 cm) that are affected by the “partial volume effect” phenomenon [9].

A recent study suggested that an SUVmax threshold of 4.85 may improve the diagnostic accuracy in differentiating benign from malignant PNs. This value is a population-specific result calculated from a total of 76 patients who met the study’s inclusion criteria and is not broadly applicable. To date, there is no accepted cut-off of SUVmax to reliably differentiate benign from malignant lesions without the need for biopsy [10]. The determination of an optimal SUVmax cut-off value for small-sized nodules requires further investigation.

SUVmean shows the average SUV within a region or volume of interest (VOI). SUVpeak reflects the average SUV in the highest metabolically active area. It is less sensitive than SUVmax and useful for therapy response assessment [4,31,42].

SUVmean is less sensitive and accurate than SUVmax. Although it can exhibit better repeatability, its test–retest reliability in clinical practice is often inferior to that of SUVpeak, and it requires standardized ROI protocols to ensure reproducibility [6]. SUVmean is now less commonly used for treatment response assessment in routine practice [43]. In conclusion, in daily clinical practice, SUVmax remains the most widely used PET metric. SUVmean provides information on the overall metabolic activity, and SUVpeak is valuable for the assessment of treatment response [11] (Table 2).

### 3.4. Novel Semi-Quantitative Metrics Beyond SUVmax—MTV and TLG

Recent research has focused on emerging semi-quantitative metrics including TLG and MTV. These parameters provide volumetric metabolic information to enhance the diagnostic performance of F18 PET/CT in indeterminate PNs [12].

Metabolic tumor volume (MTV) is a semi-quantitative PET parameter that provides a 3D assessment of the volume of tumor tissue with high metabolic activity, reflecting total tumor burden [13]. The concept of MTV in oncology was first introduced by Larson et al. in 1999 [14]. Unlike SUV, which reflects 18F FDG uptake at a specific point within a lesion, MTV quantifies the total volume of metabolically active tumor tissue. In clinical applications, SUV is used for diagnosis, risk stratification, and therapeutic management, whereas MTV is more useful for quantifying tumor burden. In detail, higher MTV values are associated with high aggressiveness and worse treatment response and prognosis. A combined approach using both SUV and MTV could hold promise for more effectively characterizing PNs [14].

Total lesion glycolysis (TLG) is another evolving quantitative PET parameter that combines metabolic activity and tumor burden by multiplying MTV with SUVmean. TLG is valuable for evaluating intratumoral heterogeneity, treatment response, and prognosis [14].

Since their initial introduction, the use of MTV and TLG has become well established in patients with Hodgkin and non-Hodgkin lymphoma. Current evidence suggests that these parameters offer greater reproducibility and improved accuracy in risk stratification [15].

Unfortunately, these metrics are not yet routinely given in PET/CT for PNs. While these findings are encouraging, further research is needed to validate their clinical utility in the assessment of patients with PNs.

Several studies focus on the evaluation of MTV and TLG parameters in patients with PNs, especially their ability to predict overall survival (OS) as well as the progression-free survival rate (PFSR) [16,17]. Multiple studies have concluded that in patients with non-small-cell lung cancer (NSCLC), elevated TLG levels are correlated with an increased mortality risk, highlighting its clinical prognostic value. On the contrary, recent data suggest that MTV has limited ability to predict OS in patients with PNs. However, the current evidence is insufficient and larger prospective studies are required to clarify the prognostic significance of both MTV and TLG [17].

Gungor et al. showed that the combined assessment of TLG and SUVmax can play a fundamental role in the diagnostic and prognostic evaluation of PNs, which is especially relevant for differentiating inflammatory from malignant nodules in patients with chronic or acute infectious diseases. They stated that TLG values in inflammatory or infectious cells are significantly lower compared to elevated SUVmax values that may mimic malignancy. On the other hand, malignant and especially the most aggressive PNs show elevated values for both SUV and TLG parameters. The authors concluded that the combined use of TLG and SUV increases both specificity and sensitivity, improving the accuracy of PN characterization and thus reducing the number of false-positive results [18].

To date, there are no accepted scoring systems for predicting the malignancy of PNs. Histopathological biopsy remains the gold-standard method for investigating PNs. Previous scoring systems, such as the Mayo model, lack precision in predicting the probability of malignancy [4]. A new scoring system called ‘LIONS PREY’ (Lung lesION Score Predicts Malignancy) was proposed by Doerr F et al. [32]. This model studied patients from a single center incorporating eight parameters (age, nodule size, spiculation, solidity, size dynamics, smoking history, pack years and cancer history). Despite the high accuracy of the scoring system (up to 95%) in predicting malignant lesions, there are several limitations such as validation across diverse populations and geographic regions and performance in real-world settings. Therefore, further well-designed studies are needed.

A recent large single-center cohort study by Pini et al. correlated SUVmax with disease stage. Patients that presented at an advanced stage demonstrated higher SUVs (SUVmax and SUVaverage), while no correlation was found between SUV and OS [6].

### 3.5. Dual-Time-Point PET/CT Imaging (DTPI)

Another approach for improving the characterization of PNs is the dual-time-point imaging (DTPI) method with 18F FDG PET/CT based on metabolic changes in 18F FDG uptake over time in nodules with different biological characteristics. This method is a subject of increasing interest among researchers. Its main goal is to improve diagnostic accuracy in the characterization of PNs, particularly in the differentiation between benign and malignant lesions.

Dual-time-point imaging involves PET/CT scanning at two time points—1 h post injection (1 h p.i) and 2 h post injection (2 h p.i)—utilizing the different FDG kinetics in malignant vs. benign nodules to assess dynamic changes in FDG uptake. In a meta-analysis, Zhang et al. confirmed the clinical impact of DTPI in reducing false-positive results. They showed that the evaluation of retention index (RI) (change in SUV between early and delayed scans) has superior diagnostic accuracy, sensitivity, and specificity compared with early PET/CT imaging alone. Malignant PNs often demonstrate increased 18F FDG uptake over time on delayed images, whereas benign PNs tend to show stable or decreasing metabolic activity [19]. Grisanti et al. confirmed that DTPI PET/CT offers additional value, especially in indeterminate PNs [20].

**Figure 2:**
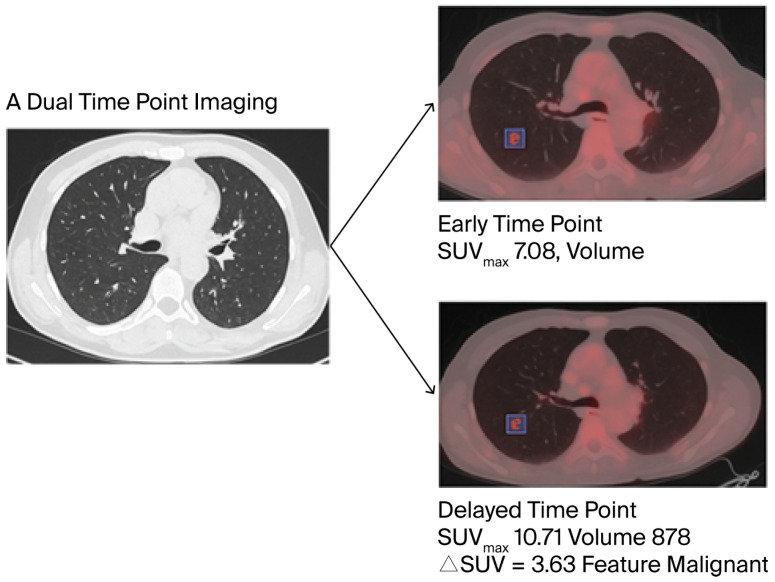
The images above demonstrate the importance of delayed imaging in differentiating benign form malignant PNs. The early-time-point image (1 h p.i) shows an SUVmax of 7.08. The delayed-time-point image (2 h p.i) shows an SUVmax value of 10.71. The change in SUVmax over time (ΔSUVmax) suggests a high probability of malignancy with a total SUVmax increase of 51%. Histopathological findings confirmed the diagnosis of lung adenocarcinoma.

Several studies showed that the RI and changes in SUVmax over time (ΔSUVmax), defined as the difference between SUVmax at the delayed and early phase (ΔSUVmax = SUVmax 2—SUVmax 1), could be additional diagnostic metrics for the accurate characterization of PNs and their malignant potential. A positive ΔSUVmax is strongly correlated with malignancy, improving risk stratification in patients with indeterminate nodules [21,22,23,24] (Figure 2).

While DTPI does not require additional radiotracer injection or specialized equipment, there are limited studies on its cost-effectiveness. Several studies have suggested that the improved diagnostic accuracy of DTPI may reduce unnecessary invasive procedures and follow-up imaging; however, there is no cost–benefit confirmation. Lin et al. and Zhang at al. show modest values for DTPI vs. single-phase imaging in the characterization of PNs [19,33,44].

In terms of availability, DTPI can be performed easily in every modern PET/CT scanner. However, despite its potential, this method has not yet been integrated in routine clinical practice.

Furthermore, DTPI is associated with several limitations and logistic challenges. The method’s delayed image acquisition, typically 90–120 min p.i, increases inconvenience to patients and complicates the routine workflow in very busy departments [21]. Furthermore, no standardized protocols or consensus is available on the optimal acquisition timing, interpretative criteria, or methods of analysis [44,45,46]. Finally, the lack of prospective multicenter studies limits the role of DTPI in routine clinical practice [19,34]. Consequently, it is recommended as an optional method for research purposes. Further multicenter prospective studies are needed to establish its cost-effectiveness and define its role in standardized diagnostic algorithms.

To evaluate the clinical value of 18F FDG DTPI PET/CT, several studies have compared this technique to histopathological biopsy as the reference method for the characterization of PNs.

Tissue biopsy remains the gold-standard method for the accurate characterization of PNs, with very high accuracy rates up to 98%. An important advantage of surgical excision biopsy includes the ability to perform a therapeutic procedure allowing complete excision (segmentectomy/lobectomy) and lymph node biopsy in a single procedure. The main methods of PN biopsy are CT-guided, bronchoscopy, and surgical excision of the lesion. CT-guided biopsy is the preferred method for peripheral lesions, with a diagnostic accuracy of up to 98%. Bronchoscopy is commonly used for central lesions and surgical excision is the method of choice for solitary PNs. The selected method mainly depends on the size and location of the PN. Although diagnosis through biopsy provides excellent results, it is an invasive procedure with side effects and complications such as pneumothorax, bleeding, and infections [25].

Moreover, sampling errors can occur especially in small or heterogenous lesions, providing false-negative results. Νon-invasive 18F FDG PET/CT biomarkers such as SUVmax, MTV, and TLG could provide similar levels of accuracy, minimizing the associated complications. Studies have shown that 18F FDG PET/CT can improve the diagnostic yield by approximately 10% and identify a safer biopsy location in 30–40% of patients [47]. These findings highlight the potential role of 18F FDG PET/CT not only in non-invasive risk stratification but also in achieving a more accurate and effective diagnosis [25,26,27,48].

## 4. Future Perspectives

Artificial intelligence (AI) and radiomics are increasingly applied to PET/CT evaluation of PNs, providing data on tumor behavior and intratumoral heterogeneity [49].

AI-based algorithms including CNN, ML, and DL techniques can automate lesion segmentation, extract radiomic features, and incorporate quantitative metrics such as SUVmax, MTV, TLG, and retention indices (RI) from DTPI, improving the diagnostic accuracy in identifying suspicious nodules and in distinguishing benign from malignant nodules [35,47,50]. Evidence suggest that AI may reduce false-positive results in the assessment of PNs [51].

Early findings suggest that integrating AI with DTPI may improve the differentiation between benign and malignant nodules, reduce interobserver variability, and support clinical decision-making. However, until now, most evidence has been based on early-phase or retrospective studies. Larger multicenter studies are required to support the integration of AI-based PET quantification into routine practice [36].

A significant development is PET/CT radiomics. This technology supports the quantitative analysis of tumors by extracting features based on heterogeneity, shape, intensity patterns, tumoral biological characteristics, aggressiveness, and prognosis, creating high-dimensional data for personalized management [37,52].

Although PET/MR offers advantages of less radiation and better soft tissue contrast, it is not a first-line imaging method for detecting and characterizing pulmonary nodules. It has several limitations, including lower sensitivity for small PNs <5 mm and non-FDG avid PNs, a long scan time, and limited availability. A systematic review by Mirshahvalad et al. reported that 18FFDG PET MR was comparable to PET/CT for detecting malignant PNs in certain cases such as pediatric patients and young adults. Moreover, quantitative PET/MR correlates well with PET/CT for large FDG PET PNs [38].

Another study by Biondetti et al. reported that PET/MR may miss small nodules [53]. PET/CT remains the gold-standard imaging method for PNs, with PET/MR playing a complementary role in research.

## 5. Conclusions

Although biopsy is the gold-standard procedure for the characterization of PNs, it is also invasive and associated with serious complications. Establishing better parameters to identify potential malignancy via PET/CT means that patients with solitary nodule disease at an early stage can be guided directly to surgery, avoiding CT FNB.

Although the heterogeneity of the studies examined indicates an inherent limitation of the literature, there is now a growing interest in more advanced, non-invasive imaging techniques with comparable diagnostic accuracy. Semi-quantitative parameters derived from 18F FDG PET/CT imaging, including SUVmax, MTV, and TLG, play a crucial role in PN evaluation. Complementary evolving parameters such as MTV and TLG appear promising in enhancing diagnostic and prognostic accuracy. Although volumetric parameters have been shown to provide additional information in characterizing the tumor burden and metabolic activity of PNs, their clinical application remains limited. There is still a lack of standardized protocols for these parameters, particularly in the work-up of PNs, which limits their routine use in everyday clinical practice.

Additionally, DTPI represents another promising approach, further improving diagnostic confidence when evaluating dynamic metabolic changes in PNs. Well-designed multicenter prospective studies are needed to standardize these imaging biomarkers for the evaluation of PN, aiming to incorporate them into clinical guidelines.

The future integration of AI may play a crucial role in advancing the clinical value of evolving PET biomarkers. The integration of AI with evolving PET biomarkers including SUVmax, TLG, MTV, and DTPI could be promising for improving diagnostic accuracy in the characterization of PNs, reducing false-positive findings, and optimizing clinical decision-making and cost-effective healthcare.

## Figures and Tables

**Table 1:** Research articles examined: type of study, number of patients, parameters evaluated, type of analysis, measurements, and results.

Authors	Type of Study	Number of Patients	Parameters	Type of Analysis	Measurement	Results
Pini et al., 2024 [6]	Retrospective	567	SUV mean SUV max	Survival analysis	Survival	HR 0.99; *p* = 0.0001
Khalaf et al., 2008 [7]	Retrospective	173	SUV max ≥ 2.5	Linear regression for different nodule sizes	Sensitivity Specificity Accuracy	For ≤1.0 cm [85%; 36%; 54%] For 1.1–2.0 cm [91%; 47%; 79%] For 2.1–3.0 cm [94%; 23%; 76%] For >3.0 cm [100%; 17%; 82%]
Lowe et al., 1994 [8]	Retrospective	93	SUR max SUR average SUR visual	ROC	AUC	SUR max 0.96 SUR avg 0.95 SUR vis 0.92
Shin et al., 2014 [9]	Retrospective	62	Resections		Accurary	32%
Ulusoy et al., 2025 [10]	Retrospective	76	SUV max	ROC	Sensitivity Specificity	93.1% cut-off 7.05 83.3 cut-off 7.05 100% cut-off 4.85 66.7% cut-off 4.85
Pellegrino et al., 2019 [11]	Retrospective	65	TLG MTV	Multivariate ROC	AUC	TLG 0.76 ΜΤV 0.73
Wang et al., 2023 [12]	Retrospective	187	Age, gender, smoking history, maximum diameter, lobulation, spike, calcification, hole, GGO status, upper lobe location of the PNs, SUVmax, SUVmean, MTV (20%), MTV (40%), TLG (20%), and TLG (40%)	Machine learning	AUC Sensitivity	86.5% 0.89
Elsadawy et al., 2024 [13]	Retrospective	40	SUVmax TLG MTV	ROC	Sensitivity Specificity	SUVmax [92%; 100%] TLG [92.3%; 100%] MTV [84.6%; 100%]
Larson et al., 1999 [14]	Retrospective	41	ΔTLG SUVmax SUVaverage	Correlation	R	ΔTLG −ΔSUVmax: 0.73 ΔTLG −ΔSUVaver: 0.78
Van Heek et al., 2022 [15]	Prospective Lymphoma	107	MTV TLG	ROC	AUC	MTV 0.69 TLG 0.69
Berghmans et al., 2008 [16]	Systematic review	1474	SUV	Meta-analysis	HR	SUV cut-off 2.08 HR 2.27 [1.70–3.02]
Wen et al., 2021 [17]	Systematic review + meta-analysis	1292	TLG MTV	Meta-analysis	HR Progression-free survival	TLG 2.02 [1.30–2.13] MTV 3.04 (1.92–4.81)
Gungor et al., 2023 [18]	Retrospective	80	SUV TLG MTV	ROC	Sensitivity Specificity AUC PPV NPV	SUV [97.6; 63.2; 0.97; 74.5; 96.0) MTV [76.2; 78.9; 0.84; 80.0; 75.0] TLG [85.7; 92.1; 0.96; 92.3; 85.4]
Zhang et al., 2013 [19]	Meta-analysis	415	18FDG-PET/CT	Pooled analysis	Sensitivity Specificity Positive likelihood ratio (LRþ) Negative likelihood ratio (LR–) Diagnostic odds ratio	Sensitivity 79% Specificity 73% PLR 2.61 NLR 0.29 Diagnostic odds ratio 10.25
Grisanti et al., 2021 [20]	Retrospective	43	RI > 10% SUVmax > 1.0 SUVmax > 1.5 SUVmax > 2.0 SUVmax > 2.5	ROC	Sensitivity Specificity PPV NPV Accuracy	RI 75.0; 73.7; 78.3; 70.0; 74.4] SUVmax > 1.0 [66.7; 26.3; 53.3; 38.5; 48.8.] SUVmax > 1.5 [33.3; 57.9; 50; 40.7; 44.2] SUVmax > 2.0 [20.8; 100; 100; 50; 55.8] SUVmax > 2.5 [25; 100; 100; 51.4; 58.1]
Matthies et al., 2002 [21]	Prospective	36	SUV	Not reported	Sensitivity Specificity	Sensitivity 0.80 Specificity 0.94
Wumener et al., 2024 [22]	Prospective	147	SUV	ROC	Sensitivity Specificity AUC	SUV [0.661; 0.870; 0.819]
Shimizu et al., 2015 [23]	Retrospective	284	SUV-E SUV-D RI	Survival	Hazard Ratio	SUV-E: HR [1.20; *p* = 0.106] SUV-D: HR [0.87; *p* = 0.117] RI: HR [4.03; *p* = 0.025]
Alkhawaldeh et al., 2008 [24]	Retrospective	265	SUV1 ≥ 2.5 SUV2 ≥ 2.5	ROC	Sensitivity Specificity Accuracy PPV NPV	SUV1 ≥ 2.5 [97; 58; 68; 46; 98] SUV2 ≥ 2.5 [84; 91; 89; 79; 93]
Li et al., 2023 [25]	Retrospective	112	CT-CNB	Logistic regression	Sensitivity Specificity	Sensitivity 97.1% Specificity 100%
Haidey et al., 2025 [26]	Retrospective	547	T-guided lung biopsy	Univariate analysis	Diagnostic rate	90.8%
Stefanidis et al., 2024 [27]	Retrospective	340	18F-FDG PET/CT	Binary regression	Probability	Overall: 83.9% Malignant:95.8%
Weir-McCall et al., 2021 [2]	Prospective	355	PET_grade_ SUV_max_, SUR_Blood_, SUV_liver_	ROC	AUC Sensitivity Specificity PPV NPV Accuracy	0.87; 87.5%; 83.6%; 87.1%; 72.2%; 80.0% 0.87; 75.6%; 84.2%; 87.3%; 70.7%; 79.2% 0.87; 72.6%; 84.9% 87.3%; 68.5%; 77.7%; 84.5%; 77.9%; 84.5%; 77.9%; 81.8%
An et al., 2022 [3]	Retrospective	665	CT-guided CNB	PMS	Accuracy Sensitivity Specificity PPV NPV	78.6%; 94.5%; 100; 96.6%
Veronesi et al., 2015 [5]	Prospective	383	PET CT	ROC	Sensitivity Specificity Accuracy	64%; 89%; 76%
Corica et al., 2023 [28]	Retrospective	146	SUVmax SUVmean MTV TLG	ROC	Sensitivity Specificity	86.6%; 69.1% 79.1%, 76.3% 74.7%; 70.9% 66%; 83.6
Nomori et al., 2004 [29]	Prospective	136	FDG-PET	ROC	Sensitivity Specificity	79%; 65%
Chen et al., 2012 [30]	Prospective	105	MTV SUV SUV_mean std_ SUV_max_ TLG	Survival	Progression-free survival Overall survival	PFS: 0% for TLG > 655 50% for TLG < 655 Overall survival: 58.8% for TLG > 655, 84.1% for TLG < 655
Shin et al., 2014 [9]	Retrospective	62	Pulmonary resection	Radiologic malignancy diagnosis	Accuracy	32%
Sugawara et al., 1999 [31]	Prospective		SUVbw SUVibw SUVlbm SUVbsa	Correlation between SUVs and weight	Pearson’s Rho	SUVbw Rho = 0.705 SUVibw Rho = −0.296 SUVlbm Rho = −0.010 SUVbsa Rho = 0.106
Larson et al., 1999 [14]	Prospective	41	DeltaTLG SUV change SUV (max)	Correlation	Pearson’s Rho	DeltaTLG with % change in SUV Rho = 0.73 DeltaTLG with SUV(max) Rho = 0.78
Doerr et al., 2024 [32]	Prospective	386	Multi-parameter model	Multivariate ROC	Classification AUC	Classification: 96% AUC: 0.94
Lin et al., 2012 [33]	Systematic review and meta-analysis	788	DTP STP	SROC	AUC	DTP: 0.839 STP: 0.757
Zhou et al., 2024 [1]	Retrospective	273	SUVmax at interval 1, 2, 3, or 4 h	Not reported	Sensitivity Specificity Accuracy PPV NPV	68.8%, 81.2%, 85.7%, and 71.4% 52.5%, 74.5%, 70.6%, and 65.0% 8.2%, 77.4%, 76.4%, and 67.6% 44.0%, 68.9%, 64.3%, and 58.8% 75.6%, 85.4%, 88.9%, and 76.5%
Barger et al., 2012 [34]	Systematic review	816	Dual-time-point FDG-PE	Meta-analysis	Sensitivity Specificity	Sensitivity: 85% Specificity: 77%
Gupta et al., 2024 [35]	Radiomics-based model	36,300	Images	SVM LASSO CNN	Accuracy AUC	SVM LASSO: 84.6%; 0.89 CNN: 98.47%; 0.998
Liu et al., 2025 [36]	Retrospective	311	Ground-glass nodules	CNN	Accuracy Specificity Sensitivity AUC	84.8% 84.6% 84.9% 0.85
Salihoğlu et al., 2022 [37]	Retrospective	48	18F-FDG PET/CT scan	Deep learning	Sensitivity AUC	88% 0.81
Mirshahvalad et al., 2023 [38]	Systematic Review/meta analysis	1278	MRI, 18F-FDG PET/MRI	Hierarchical summary ROC	Sensitivity Specificity	MRI: 96%; 100% ^18^F-FDG PET/C: 99%; 99%

**Table 2:** Types of SUVs.

Types of Standardized Uptake Values (SUVs) in FDG PET/CT	
By normaliztion	Body weight SUV (SUVbw)
	Lean body mass SUV (SUVlbm)
	Body surface area SUV (SUVbsa)
By measurement	SUVmax
	SUVmean
	SUVpeak
Novel semi-quantitative metrics	MTV
	TLG

## Data Availability

Data are contained within the article.

## Abbreviations

The following abbreviations are used in this manuscript:

PN	pulmonary nodule
18F FDG	18 Fluorodeoxyglucose
PET/CT	positron emission tomography/computed tomography
SUV	standardized uptake value
SUVmax	standardized uptake value maximum
SUVmean	standardized uptake value mean
SUVpeak	standardized uptake value peak
MTV	metabolic tumor volume
TLG	total lesion glycolysis
DTPI	dual time point imaging
ΔSUV	SUV2 (delayed phase) -SUV1 (early phase)
ΔMTV	MTV2 (delayed phase) -MTV1 (early phase)
ΔTLG	TLG2 (delayed phase) -TLG1 (early phase)
WHO	World Health Organization
ROI	region of interest
OS	overall survival
CT	computed tomography
GLUT	glucose transporter
AI	
PETMR	
Radiomics	
